# Real time monitoring of transtibial elevated vacuum prostheses: A case series on socket air pressure

**DOI:** 10.1371/journal.pone.0202716

**Published:** 2018-10-22

**Authors:** Katherine R. Schoepp, Jonathon S. Schofield, David Home, Michael R. Dawson, Edmond Lou, McNiel Keri, Paul D. Marasco, Jacqueline S. Hebert

**Affiliations:** 1 Department of Medicine, Faculty of Medicine and Dentistry, University of Alberta, Edmonton, Alberta, Canada; 2 Department of Biomedical Engineering, Lerner Research Institute, Cleveland, Ohio, United States of America; 3 Prosthetics and Orthotics Department, Glenrose Rehabilitation Hospital, Edmonton, Alberta, Canada; 4 Department of Electrical & Computer Engineering, Faculty of Engineering, University of Alberta, Edmonton, Alberta, Canada; 5 Advanced Platform Technology Center of Excellence, Louis Stokes Cleveland Department of Veterans Affairs Medical Center, Cleveland, Ohio, United States of America; Holland Bloorview Kids Rehabilitation Hospital, CANADA

## Abstract

Prosthetic elevated vacuum is a suspension method used to reduce daily volume changes of the residual limb. Evaluation of the effectiveness of these systems is limited due to a lack of correlation to actual socket air pressure, particularly during unconstrained movements. This may explain some of the variability in functional outcomes reported in the literature. Our objective was to develop a light-weight portable socket measurement system to quantify internal socket air pressure, temperature, and acceleration; and to present preliminary results from implementation with three transtibial prosthesis users with mechanical elevated vacuum pumps. Participants completed five functional tasks with and without the vacuum pumps actively connected, including the 2-Minute Walk test, 5-Times Sit-to-Stand test, 4-Square Step test, L-Test, and Figure-8 test. Results demonstrated different gait profiles and pressure ranges for each user. Two of the participants demonstrated substantially lower air pressure (higher vacuum) over time while the pump was active compared to inactive. The minimum air pressure measured for all participants was -34.6 ± 7.7 kPa. One participant did not show substantial changes in pressure over time for either pump condition. Functional task performance was not significantly different between pump conditions. Correlation with accelerometer readings indicated peak positive pressures occurred just following initial contact of the foot in early stance, and the most negative pressures (highest vacuum) were observed throughout swing. This study has demonstrated the use of a portable data logging tool that may serve the clinical and research communities to quantify the operation of elevated vacuum systems, and better understand the variability of mechanical pump operation and overall system performance.

## Introduction

In 2005, there were an estimated 1.6 million people living with an amputation in the United States; this number is expected to increase to 3.6 million by 2050 [1]. Despite advances in prosthetic limb development, optimal socket fit remains a challenge [2–4]. Poor suspension may result in slippage between the socket and the residual limb, particularly during the cyclical loading and unloading associated with gait, which can compromise stability [2]. This can promote irritation, discomfort, and tissue damage [5]. One approach to minimizing this slippage is using elevated vacuum suction suspension, where sub-atmospheric pressure (vacuum) is employed to reduce the relative movement of the user’s residual limb with their prosthetic socket [6]. In a typical elevated vacuum socket, the residual limb is covered by a gel liner which sits within a rigid prosthetic socket, and a vacuum is applied through a one-way valve to the space between these layers to improve their connection. The connection between the liner and prosthetic socket is maintained using a proximal seal, which is typically either a suspension sleeve or inner sealing gasket (6). Elevated vacuum systems are predominantly used for attaching lower-limb sockets, though recently there has been preliminary work showing promise for use in transradial [7] and partial-foot amputation cases [8].

Several studies have demonstrated benefits in using elevated vacuum in lower-limb prostheses. When compared to passive suction sockets, vacuum pumps have been shown to maintain or increase residual limb volume during gait [9,10]. This may be due to changes in socket-limb interface pressure, where the vacuum reduces positive contact pressures during stance and increases negative air pressures during swing, thereby increasing the fluid drawn into the limb [11]. In support of this theory, bioimpedance analysis demonstrated an increase in extracellular fluid volume when walking using a transtibial prosthesis with elevated vacuum [12]. Residual limb movement relative to the socket (i.e. pistoning) has been shown to be lower when using elevated vacuum compared to traditional suction and pin-locking systems, with increasing vacuum pressures correlated to reduced pistoning [10,13–15]. Improved balance and gait when using elevated vacuum systems has also been demonstrated [10,16,17]. Compared to pin-locking and traditional suction sockets, elevated vacuum has demonstrated improved perfusion and preservation of skin barrier function after 16 weeks of use [18]. In fact, several studies have found that elevated vacuum systems do not preclude wound healing, and allow patients to ambulate sooner and for longer periods of time compared to other systems [19–21]. Generally, elevated vacuum systems are viewed favourably by clinicians, however questionnaire results have shown that they are perceived as being “more expensive, heavier, less durable, and require more maintenance” than a standard socket [22]. Several review articles have been published in this area, and while existing evidence for elevated vacuum systems is promising, these reviews have indicated a need for more controlled studies, larger sample sizes, and evaluation of long-term effects [6,22–24].

Recent findings have shown that the level of vacuum (i.e. negative air pressure) is directly related to the amount of pistoning [13], and that changes in pressure may be related to quality of socket fit [25]. However, many studies regarding the effectiveness of elevated vacuum do not monitor socket air pressure. Bench-top testing of both electrical pump systems [26,27] and mechanical elevated vacuum systems [26] highlight model-specific differences in measures of performance such as maximum gauge pressure and air evacuation time [26,27]. These differences may help to explain variability in study findings, such as in the case series by Sanders *et al*. that found inconsistent results across different elevated vacuum systems [12]. Monitoring vacuum pressures while wearing a prosthesis with elevated vacuum could possibly shed light on these differences. For in-lab testing, a pressure monitor (model 2L760, DigiVac, Matawan, NJ) has been used to quantify socket air pressure [26–28]. Because this system is tethered to a computer system and comes with the cost of increased bulk and weight it may not appropriate for tasks that require free movement, limiting its use to standing, sitting, and treadmill walking. Xu *et al*. (2017) developed a pressure measurement system to induce a specific vacuum level in order to study the effect on gait parameters, but did not report the changes in vacuum pressure throughout the trials [29]. The LimbLogic VS Communicator (Ohio Willow Wood) has been developed to measure socket air pressure in real-time [13,25,30], however it is only designed to interface with the LimbLogic VS system, limiting its usability across a wider range of systems. A discrete monitor that could be used across elevated vacuum systems to measure and log socket air pressure in real-time across of variety of functional tasks could provide valuable quantitative comparisons.

To address these limitations, we developed a light-weight portable socket measurement system capable of capturing internal socket air pressure, temperature, and acceleration. Temperature and acceleration measurements were included to provide insight as to whether socket temperature or movement may impact internal air pressure. Acceleration measurements can also be used to temporally align pressure readings with the gait cycle. This system can either log data to onboard memory, or stream wirelessly and in real-time to a computer. The objective of this paper is to describe the system design, fabrication, and integration of the device, as well as present preliminary results from implementation with three transtibial prosthesis users with mechanical elevated vacuum pumps.

## Methods

Ethics approval was obtained through the University of Alberta’s institutional review board and participants gave written informed consent prior to participation. Participant recruitment was based on a convenience sample. Inclusion criteria were adults (18 to 75 years of age) with a major lower limb amputation (at or above the ankle), wearing a lower limb prosthesis with a vacuum seal in the socket, and at least K-level 3 (unlimited community ambulator). Exclusion criteria were any skin, pain, or balance conditions that would preclude the ability to wear a prosthetic socket, or cognitive impairments or language barriers precluding providing informed consent or responding to survey questions. Three participants currently using an elevated vacuum system in their prosthesis were recruited through prosthetics shops, with details listed in Table 1. Each prosthesis was evaluated by a certified prosthetist and was deemed to be well-fitting at the time of testing. To confirm quality of fit, participants completed the OPUS Lower Extremity Functional Status Measures survey [31], with results ranging from 50 to 70 out of a total possible score of 80. Note that “short” limb length refers to a tibia length less than 12 cm and “medium” is between 12 and 15 cm [32].

**Table 1:** Participant information and prosthetic components.

	Participant 1	Participant 2	Participant 3
*Participant information*			
Sex	Male	Male	Male
Weight	225 lbs	175 lbs	210 lbs
Height	5’11”	5’10”	6’2”
*Amputation information*			
Amputation level	Transtibial	Transtibial	Transtibial
Side of amputation	Right	Left	Right
Type of amputation	Trauma	Vascular	Vascular
Time since amputation	4 years, 1 month	3 years, 4 months	8 years, 4 months
Limb length, geometry	Short, Conical	Medium, Cylindrical	Medium, Cylindrical
*Wear and performance*			
Hours per day prosthesis worn	16	16	16
Days per week prosthesis worn	7	7	7
OPUS Functional Status Score	50 out of 80	70 out of 80	69 out of 80
*Prosthetic components*			
Elevated vacuum system	Harmony P3 (Ottobock, 4R147 = K)	Unity Sleeveless Vacuum (Össur)	Triton H (Ottobock, 1C62, Rt. Cat. 3-4-P4N)
Liner	Alpha Design Custom Liner (WillowWood, ALC-DES-EO)	Alpha Classic Liner (WillowWood, ALC-5064-E)	Anatomic 3D PUR Liner (Ottobock, 6Y512 = 265x125-F)
Foot	LP Vari-Flex (Össur, 27R C7)	Pro-Flex LP Torsion (Össur, PLTO425L)	Triton H (Ottobock, 1C62, Rt. Cat. 3-4-P4N)
Sleeve	Extreme Sleeve (Alps, SFK-28-3)	ProFlex Plus Sleeve (Ottobock, 453A = 1–0)	ProFlex Plus Sleeve (Ottobock, 453A40 = 2–7)
Socket type	Thermoplastic temporary socket	Laminated	Environmentally Managed System, Laminated (EMS)

### Design and installation of data logger

The socket data logger was developed in-house and contained three sensors; an air-pressure sensor (MPXx6250A, Freescale Semiconductor, range: 20 to 250 kPa absolute, reported accuracy: ± 0.25 kPa), an external temperature probe (LMT86, Texas Instruments, range: -50 to 150°C, reported accuracy: ± 0.4°C), and an inertial measurement unit (MPU-9250, InvenSense, range: -8 to 8 G, reported accuracy: ± 0.05 G). Overall dimensions of the device including housing were 18 x 38 x 51 mm with a total weight of 27 g. The device was powered by a lithium polymer battery and communicated using Bluetooth LE in real-time via a custom graphical user interface (GUI) at a frequency of 25 Hz while, simultaneously logging the data to internal on-board memory at the same rate for redundancy. Benchtop validation testing using the Harmony e-pulse (Ottobock, Germany) connected to a sealed empty chamber (approximately 130 cm^3^) indicated minimum evacuated pressures of -62.8 ± 1.4 kPa, consistent with measurements in literature [26,27].

**Fig 1:**
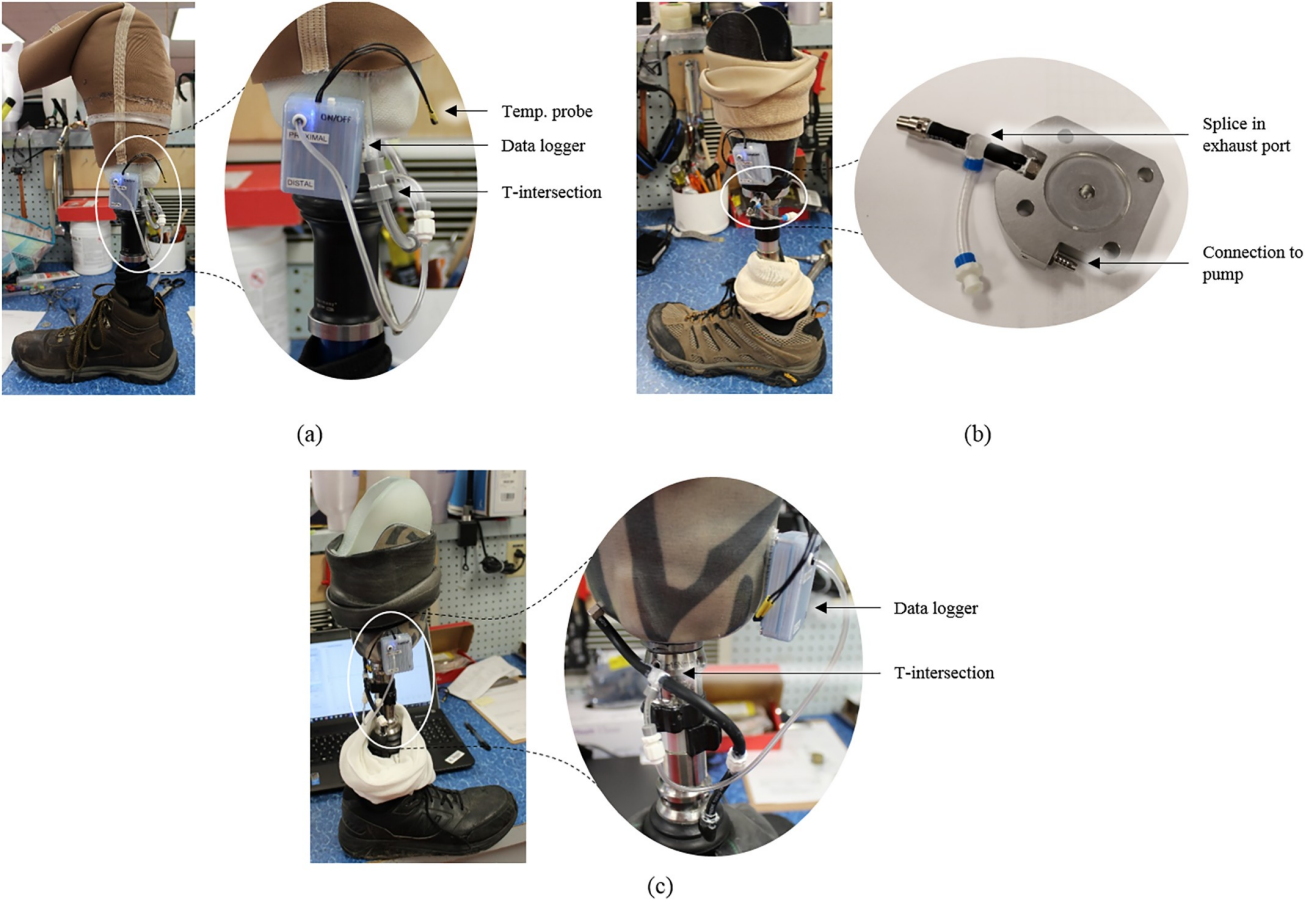
Installation of data logger onto prosthetic socket. Modified sockets for (a) Participant 1, (b) Participant 2, and (c) Participant 3.

Air pressure measurements in the socket were obtained by connecting the sensor to the socket via the existing tubing (Participants 1 and 3) or exhaust port connector (Participant 2) between the pump and socket, shown in (Fig 1). A narrow tubing diameter was selected (1/16 inches inner diameter) to ensure that the inclusion of the data logger would have minimal impact on the overall volume of the prosthetic socket; the volume increase for a 20 cm length of tubing is 0.4 cm^3^, relatively small compared to estimated socket volumes ranging from 33 to 197 cm^3^ [27]. Therefore, consistent with Boyle’s law and previous prosthetic literature [30], air pressure measurements within this additional tubing are equivalent to the air pressure throughout the prosthetic socket. The temperature probe was placed on the outside of the socket and covered with the prosthetic liner. The housing containing the inertial measurement unit was mounted to the outside of the rigid socket, as was the temperature probe.

Pump performance was evaluated in two conditions; active and inactive. In the active condition, the pump was connected to the socket as per manufacturer’s instructions. In the inactive condition, the connection to the pump was replaced by a plug, thereby separating the pump from the socket. Trials were double-blinded; both the participant and researchers did not know the condition of the pump. To ensure the double-blind condition was maintained, a certified prosthetist was responsible for connecting or disconnecting the pump between trials and did not communicate the state of the system until after data analysis was complete. A shroud was placed over the entire prosthetic leg to hide any visual clues. There were however minor differences in auditory cues in the different pump conditions.

### Functional tasks and subjective surveys

Five mobility tasks were performed for each trial in the same order. Tasks were selected to capture different movements representative of everyday life, including walking, sit-to-stand, turning, and stepping. The first task was a 2-Minute Walk Test, similar to [33], where the participant walked in a large circular hallway (circumference of 190 m) for two minutes. The total distance travelled was measured using a measuring wheel (Rolatape Measuring Systems, Model MM-45M). The participant then completed the Five Times Sit-to-Stand test [34], the 4-Square Step test [35], the L-Test [36], and the Figure-8 test [37]; time to task completion, number of steps, and errors were determined from analysis of video footage. At the beginning of the test session, task instructions were provided to the participant and they were given the opportunity to practice each task until comfortable with their performance. This was done to minimize potential learning effects during the trials. During each trial, the 2-Minute Walk test and 5-Times Sit-to-Stand test were completed once, and the 4-Square Step test, L-Test, and Figure-8 test were completed twice.

At the beginning of each trial, the prosthetist connected or disconnected the pump in a room separate from the participant and researchers. Each condition (pump active or inactive) was evaluated twice for a total of four trials, with order of condition block randomized in pairs. Participants were asked to don their prosthesis as usual, then they completed the functional tasks outlined in the order above. If a mistake was made during one of the functional tasks, that specific task was repeated immediately. Once the functional testing was complete, participants were asked to doff their prosthesis, and a seated break of at least five minutes was enforced prior to the next trial.

After each trial the participant completed a short survey to capture their impressions of the prosthesis under the current condition. The survey was modified from the OPUS Satisfaction with Device Score [38], and used a 5-point Likert scale, from 1 (strongly disagree) to 5 (strongly agree), shown in S1 Table.

### Data treatment

Analysis was conducted using Excel (Microsoft, 2016) and Matlab (Mathworks, R2017b). Gauge pressure data and acceleration magnitudes were analyzed. For each of the activities, data was broken into individual movements of the gait cycle (i.e. strides) by delineating at maximum pressures within the approximate stride period specified; note that data segments were shifted forward by 10 timesteps (0.4 s) to visually capture the data surrounding the peaks in pressure. The exception is for the sit-to-stand motion where minimum pressures were used to separate movements. The data was then normalized over movement length, creating a scale of 0 to 100% movement completion that allowed for the data to be plotted by condition. Pressure change over time was calculated by determining the slope of the data using simple linear regression. From each individual movement, average and standard deviation values were determined. Two-sample t-tests were used to evaluate differences between active compared to inactive conditions, with variance conditions confirmed using f-tests, and α = 0.05. Note that Trial 4 from Participant 3 was excluded from the analysis due to technical challenges resulting from the donning process that resulted in inadequate seal from the sleeve, compromising the suction suspension.

## Results

Data analysis was completed for each of the five functional tasks. A detailed description of the 2-Minute Walk test is presented, as the large number of cyclical loading and unloading movements provided the most detail. A summary of the results from the remaining tests is then provided. Finally, functional task performance and qualitative survey responses are presented.

### Detailed analysis of 2-Minute Walk test

**Fig 2:**
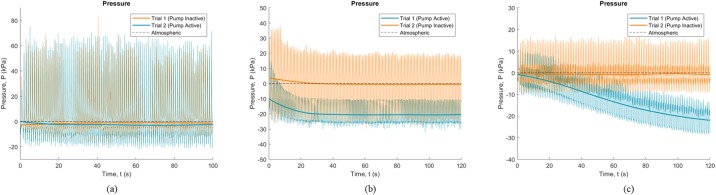
Overall gauge pressure data during 2-Minute Walk test. Data is shown across (a) Participant 1, (b) Participant 2, and (c) Participant 3. Thin lines indicate raw data and thick lines indicate measurements smoothed using the rloess function in Matlab.

Gauge pressure data collected during the 2-Minute Walk test for each participant are shown in Fig 2. The thin lines indicate the real-time pressure measurements, which fluctuated substantially with each stride. The thick lines indicate the overall change in pressure. For Participant 1 (Fig 2a), the pressure remained fairly consistent, regardless of pump condition. For Participant 2 (Fig 2b), in both conditions the pressure dropped (vacuum increased) initially, then stabilized to different values depending on pump condition. For Participant 3 (Fig 2c), the pressure was fairly consistent when the pump was inactive and fell continuously when the pump was active.

**Fig 3:**
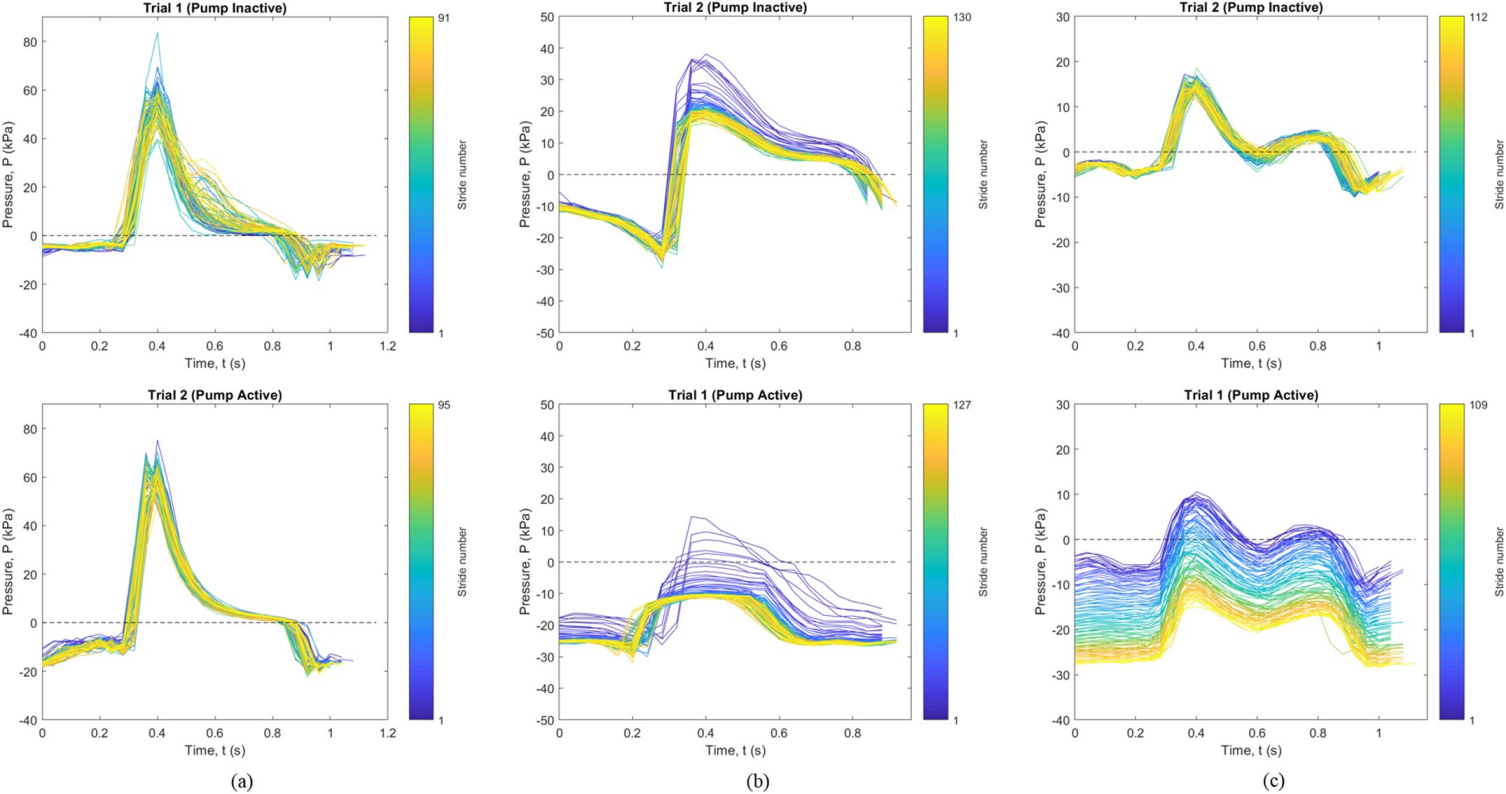
Individual gauge pressure stride data during 2-Minute Walk test. Data is shown with pump inactive (top) and pump active (bottom), across (a) Participant 1, (b) Participant 2, and (c) Participant 3. Blue indicates the first stride and yellow the last, with the legend indicating total stride count.

Data for the 2-minute walk test was broken into individual strides as shown in Fig 3. Early strides are indicated in blue with later strides in yellow. This visualization demonstrates differences in vacuum pressures over time. In the case of Participant 1 (Fig 3a), the use of elevated vacuum (pump active) appeared to reduce the variations in pressure occurring with each stride, but not the overall pressures. For Participant 2 (Fig 3b) there was a reduction in overall gauge pressure (increase in vacuum) with subsequent strides in both conditions, with more consistently negative vacuum pressures with the pump active. For Participant 3 (Fig 3c), there was a clear effect of the active pump condition showing progressive reduction in pressures with subsequent strides, compared to the no pump condition which show little to no change.

**Fig 4:**
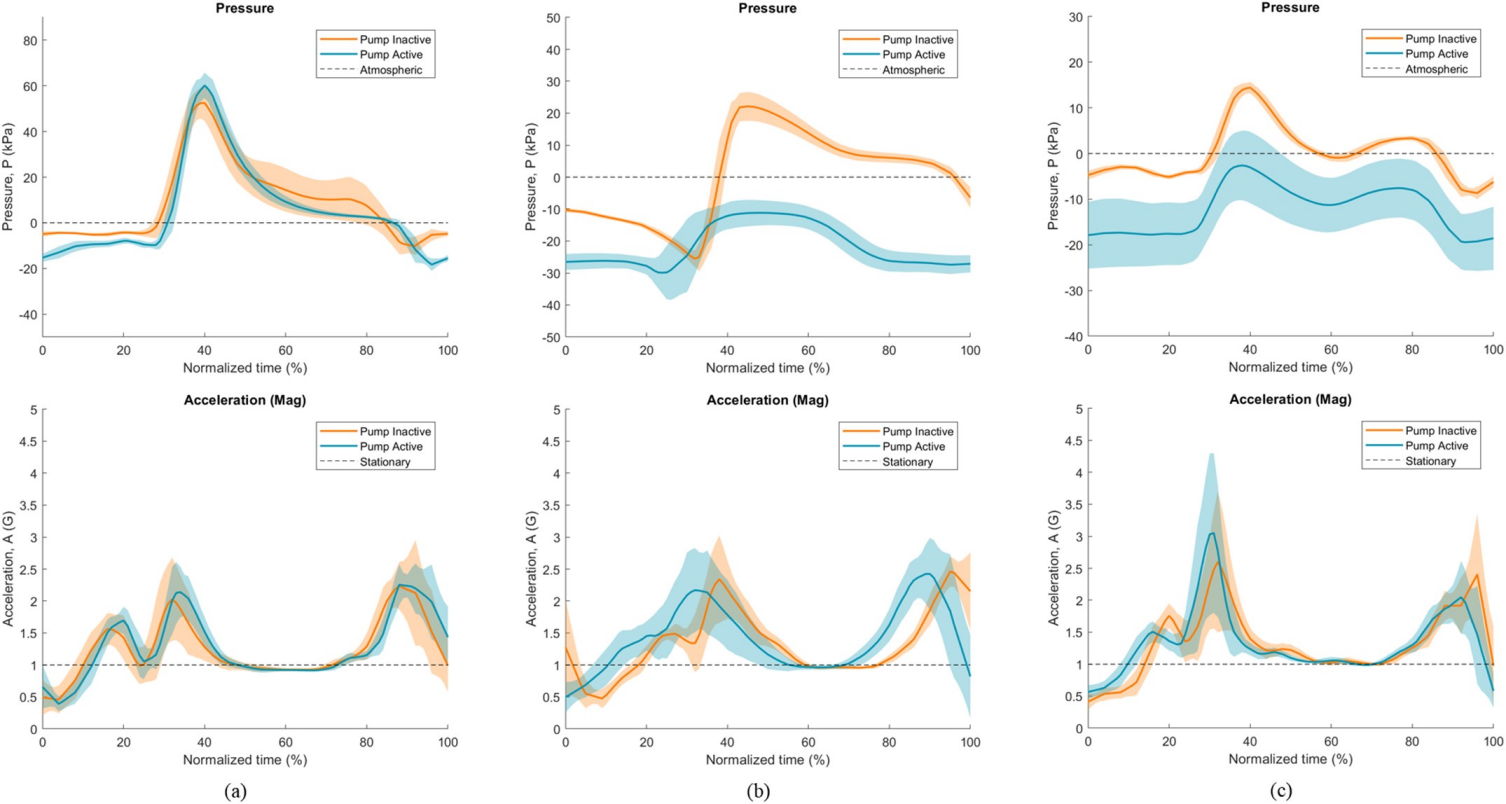
Normalized individual stride data during 2-Minute Walk test. Data is shown for gauge pressure (top) and magnitude of acceleration (bottom), across (a) Participant 1, (b) Participant 2, and (c) Participant 3. Dark lines indicate average measurements, with shaded areas indicating standard deviation.

Average gauge pressure and acceleration data for the 2-Minute Walk test are shown in Fig 4, normalized over the full stride length. As above, Participant 1 (Fig 4a) visually showed small differences in absolute pressure between conditions, and lower pressure variation with the pump active. Participants 2 (Fig 4b) and 3 (Fig 4c) showed large differences in pressure between conditions, where the active elevated vacuum reduced overall pressure. Across all three participants, peaks in pressure were followed by drops; these drops coincided with stable acceleration measurements, where acceleration magnitude was close to 1.0 G.

Values and statistical results from the data analysis of gauge pressure and acceleration are presented in Table 2. Gauge pressure was statistically lower (vacuum higher) while the elevated vacuum system was active, though the effect size varied by participant. Pressure change over the duration of the walk was significantly different for both Participants 1 and 3, where the active pump condition showed an overall reduction in socket pressure. There significant differences in acceleration magnitude between conditions for Participants 1 and 2, though effect size may not be clinically significant.

**Table 2:** Average measured results over individual strides during 2-Minute Walk test. Reported as mean ± standard deviation. Significant differences are highlighted. Abbreviations are as follows: press. (pressure), accel. (acceleration), mag. (magnitude).

		Participant 1			Participant 2			Participant 3		
		Inactive	Active	P-value	Inactive	Active	P-value	Inactive	Active	P-value
2-Minute Walk	Gauge press. (kPa)	8.5 ± 2.9	4.6 ± 1.3	< 0.001	-0.1 ± 1.4	-21.7 ± 3.2	< 0.001	0.1 ± 0.3	-12.4 ± 6.7	< 0.001
	Accel. mag. (G)	1.2 ± 0.1	1.3 ± 0.0	< 0.001	1.3 ± 0.1	1.4 ± 0.1	< 0.001	1.3 ± 0.1	1.3 ± 0.1	0.743
	Press. change (kPa / min.)	1.6 ± 0.3	-0.5 ± 0.2	0.016	-1.6 ± 0.0	-2.6 ± 0.5	0.096	0.1 ± 0.2	-11.7 ± 0.0	0.014

For further insight into differences with vacuum pressures during the 2-Minute Walk test, we analyzed the first 5 and last 5 strides of the task (Table 3). In all instances, there were significant differences in gauge pressure between the active versus inactive conditions. Differences in pressures between the active and inactive conditions were more pronounced for the last 5 strides of the test compared to the first 5 strides, particularly for Participants 2 and 3. Notably, the maximum air pressure for Participant 2 and 3 for the last 5 strides of the task with the pump active maintained negative values, meaning that the socket was sub-atmospheric throughout the entire gait profile, in contrast to the positive values in the inactive pump condition.

**Table 3:** Average gauge pressure data across initial and final strides during 2-Minute Walk. Reported as mean ± standard deviation. Significant differences are highlighted.

		Participant 1			Participant 2			Participant 3		
		Inactive	Active	P-value	Inactive	Active	P-value	Inactive	Active	P-value
Initial 5 strides	Min. press. (kPa)	-13.8 ± 2.0	-18.5 ± 1.2	< 0.001	-24.9 ± 1.9	-28.9 ± 5.3	0.022	-9.8 ± 0.7	-10.1 ± 0.3	0.005
	Max. press. (kPa)	58.1 ± 10.9	66.7 ± 7.2	0.035	38.6 ± 3.6	4.3 ± 5.2	< 0.001	15.0 ± 0.9	9.6 ± 0.6	0.001
Final 5 strides	Min. press. (kPa)	-12.9 ± 1.8	-20.5 ± 1.7	< 0.001	-27.5 ± 3.2	-34.6 ± 7.7	0.002	-9.3 ± 0.4	-28.3 ± 0.1	< 0.001
	Max. press. (kPa)	54.5 ± 3.4	61.8 ± 4.3	0.007	20.0 ± 1.7	-12.2 ± 1.8	< 0.001	15.7 ± 0.8	-13.5 ± 1.0	< 0.001

### Analysis of remaining functional tests

Values and statistical results from the data analysis of gauge pressure and acceleration for the remaining four tasks are presented in Table 4. Differences between pump active and inactive were smaller during the shorter duration tasks compared to the 2-Minute Walk test.

**Table 4:** Average measured results over individual strides during remaining functional tests. Reported as mean ± standard deviation. Significant differences are highlighted. Abbreviations are as follows: press. (pressure), accel. (acceleration), mag. (magnitude).

		Participant 1			Participant 2			Participant 3		
		Inactive	Active	P-value	Inactive	Active	P-value	Inactive	Active	P-value
5 Times Sit-to-Stand	Gauge press. (kPa)	2.7 ± 0.7	0.6 ± 0.4	< 0.001	-0.7 ± 0.5	-4.0 ± 2.9	0.042	-1.8 ± 1.1	-10.1 ± 1.0	< 0.001
	Accel. mag. (G)	1.0 ± 0.00	1.0 ± 0.01	0.193	1.0 ± 0.00	1.0 ± 0.01	0.112	1.0 ± 0.01	1.0 ± 0.00	0.303
4-Square Step Test	Gauge press. (kPa)	5.7 ± 4.0	6.6 ± 2.9	0.264	1.3 ± 5.0	-5.7 ± 4.7	< 0.001	0.8 ± 1.0	-4.5 ± 1.2	< 0.001
	Accel. mag. (G)	1.2 ± 0.13	1.1 ± 0.03	< 0.001	1.2 ± 0.12	1.1 ± 0.11	0.311	1.1 ± 0.05	1.1 ± 0.04	0.919
L-Test	Gauge press. (kPa)	9.3 ± 3.3	7.0 ± 2.9	< 0.001	7.0 ± 6.2	-11.0 ± 5.6	< 0.001	0.9 ± 0.7	-4.3 ± 1.1	< 0.001
	Accel. mag. (G)	1.3 ± 0.16	1.2 ± 0.07	< 0.001	1.3 ± 0.12	1.3 ± 0.13	0.521	1.2 ± 0.08	1.2 ± 0.10	0.207
Figure-8 Test	Gauge press. (kPa)	7.7 ± 4.6	6.0 ± 1.9	0.149	3.4 ± 3.4	-9.3 ± 3.5	< 0.001	0.2 ± 0.6	-2.9 ± 0.9	< 0.001
	Accel. mag. (G)	1.3 ± 0.16	1.3 ± 0.12	0.433	1.2 ± 0.11	1.3 ± 0.10	0.282	1.2 ± 0.06	1.2 ± 0.07	0.586

Temperature measurements of the external socket ranged between 22 and 27°C, however there was no correlation to pump condition.

### Functional task performance

Functional task performances are summarized in S2 Table. There were no significant differences in performances based on pump condition (*p* > 0.05 for all comparisons). Functional task performance in both conditions and across all participants fell within normative walking distances during the 2-Minute Walk test [33]. Performance during the L-Test and Figure-8 test exceeded reported values based on populations of transtibial amputees and people with mobility disabilities, respectively [36,37]. Task durations of the 5 Times Sit-to-Stand and 4-Square Step tests were longer than values reported in normative adult populations [35,39].

### Qualitative survey responses

**Fig 5:**
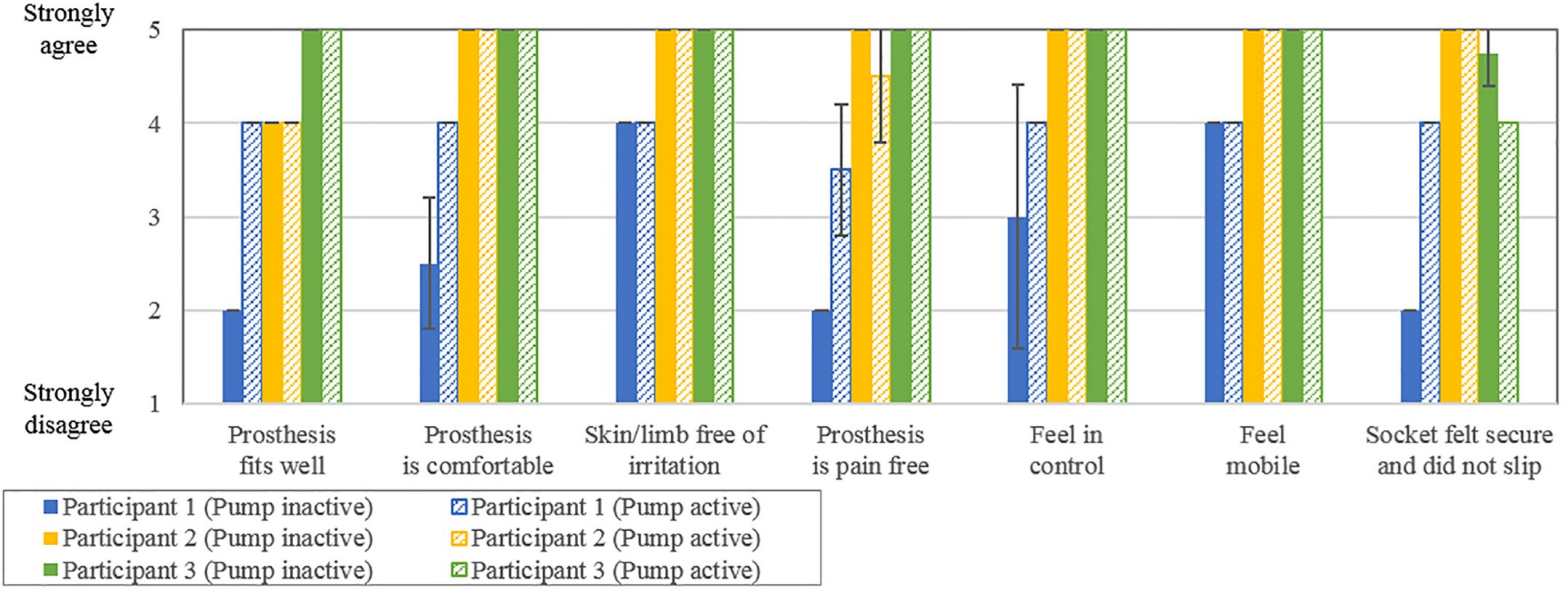
Comparison of qualitative survey scores. Solid colour bars indicate inactive pump condition scores and hatched bars indicate active pump condition scores, with error bars indicating standard deviation.

Responses to the qualitative survey are summarized in Fig 5. Despite our attempts to blind the participants to the condition of the pump, Participant 1 was able to correctly identify pump condition in all trials. Survey responses of Participant 1 indicated a preference towards the use of the elevated pump, with a perceived improvement in prosthesis fit and comfort, reduced pain and perception of slippage, as well as a greater feeling of control. Participant 2 misidentified the pump conditions for Trials 1 and 2 and was correct for Trials 3 and 4. Participant 3 misidentified every pump condition. Survey responses from Participants 2 and 3 did not indicate a clear preference towards either pump condition.

## Discussion

We have developed and applied a device that is able to capture real-time socket pressure and acceleration data while worn non-intrusively and without restricting mobility. This study has demonstrated the performance of the data logger and allowed for the evaluation of air pressure within three different mechanical elevated vacuum systems while worn and performing standardized functional mobility tasks. The use of the pumps resulted in significant changes to socket air pressure over time, where each participant demonstrated different gait profiles and pressure ranges.

With the pump active, both Participants 2 and 3 demonstrated a substantial decrease in socket air pressure over the duration of the 2-Minute Walk test. For Participant 2, the air pressure dropped then plateaued after approximately 50 steps. However, for Participant 3, the pressure dropped continually over the duration of the trial. The air pressure in both participants’ sockets reached a similar final negative pressure range at the end of the task. With the pump inactive, the air pressure values at the end of the trials were similar to those at the beginning; the maximum pressure continued to fluctuate above atmospheric pressure. However, with the pump active, air pressure readings were consistently negative for the final 5 strides, indicating that the elevated vacuum was maintaining a sub-atmospheric pressure throughout the full stride. Interestingly, these two participants had difficulties distinguishing when the pump was active, and questionnaire results did not show a clear preference towards either condition. It was surprising that the participants with apparently effective vacuum systems could not accurately detect when the pump was active.

In contrast, the air pressure in Participant 1’s socket did not change substantially over the 2-Minute Walk test, in both active and inactive pump conditions. However, with the pump active there was a more consistent pressure profile. This participant was the most successful at correctly identifying pump condition, and questionnaire results indicated a strong preference towards the pump being active. Differences between participants may be due to a combination of factors other than pump design, including socket fit and material properties, limb geometry, donning process, and gait pattern, to name a few. In particular, this participant had a short conical limb with less soft tissue coverage, in comparison to the other 2 participants. Future work should investigate the effect of soft tissue compliance and volume on effectiveness of vacuum systems.

Similar pressure trends were seen during the other walking-based tasks (Figure-8 and L-test), though they were not as pronounced due to the shorter task duration. Some average acceleration magnitudes were significantly different between conditions; however, these differences were very small and may not be clinically relevant. The 5-Times Sit-to-Stand activity yielded lower variance in pressure and acceleration compared to other activities, likely because the prosthetic leg was planted against the floor rather than suspended from the limb in swing phase.

Using the measured acceleration readings, changes in socket air pressure can be roughly correlated to phases of the gait cycle. Willemsen *et al*. demonstrated that stable accelerations equivalent to gravity correspond to stance, variable readings to swing, and that peaks occur during push off and foot down [40]. We can therefore infer that peak positive pressures occurred just following initial contact of the foot in early stance, with pressure decreasing during stance. The most negative pressures (highest vacuum) were observed throughout swing. This is similar to the pressure profiles measured by Chino *et al*., where nine transtibial amputees using suction sleeves were evaluated [41]. It may be valuable in future work to synchronize the pressure profiles with specific phases of the gait cycle.

Pressure ranges reported in literature vary depending on the type of pump used. Our measured results did not attain negative pressures as low as the benchtop testing conducted by Komolafe *et al*. [27], which evaluated different mechanical pumps using a material testing system and found that a vacuum pressure of -57.6 kPa could be achieved in less than 50 loading cycles or 80 seconds. Xu *et al*. [29] recommended a moderate level of 50 kPa to optimize comfort and gait symmetry; their pressures were manually pulled rather than induced by the mechanical pump. The minimum pressures observed at the completion of the 2-Minute Walk test during our study were -34.6 ± 7.7 kPa. This may be due to differences between idealistic ‘bench-top’ testing conditions and real-world prosthetic sockets worn by participants; loading profiles and rates were substantially different, and it is likely that the seal of a socket on an amputated limb is inferior to an idealized system. Chino *et al*. found minimum pressures using a suction sleeve ranged between -7 and -31 kPa over ten gait cycles [41]. In contrast, air pressures created by electrical elevated vacuum pumps have been reported to range between -27 to -85 kPa [9–11,13,19,20,25,26,28,30]. This disparity indicates there may be large differences in air pressure between systems, and more evaluation is needed to better understand the impact of these differences on prosthesis user function.

Functional task performances were compared to reported data. All three participants met or exceeded reported scores for the walking-based tasks (2-Minute Walk, L-Test, and Figure-8) but demonstrated reduced performance for both the 5 Times Sit-to-Stand and 4-Square Step tests. There were no significant differences in task performances between pump conditions, suggesting that the short-term use of elevated vacuum may not have a measurable impact on functional mobility. This is in contrast with the more longer term study conducted by Samitier *et al*. [17] that found improvements in functional task scores of 16 transtibial participants after 4-weeks of training with an elevated vacuum system, when compared to their previous system.

### Limitations and future work

This study quantified socket air-pressure across three elevated vacuum systems within worn prosthetic sockets, during specific tasks allowing unconstrained movement in the clinical lab environment. The data logger tool, testing protocols, and analyses presented contribute to the clinical and research communities by helping to quantify the operation of elevated vacuum systems, and to bridge the gap between the measurement of mechanical pump operation and overall system performance and function.

The main limitation of this study is that it is a case series where the elevated vacuum components and fittings were not controlled; while this inherently makes it difficult to draw specific conclusions, the large variability between users has demonstrated the need to question whether or not these systems work effectively in all users. Future studies should be controlled to allow for specific conclusions to be drawn.

The short duration of the study is also a limitation; the next step is to study trends over longer periods of time. Collecting the data within a clinical lab environment may have affected the participants’ use of their prosthesis; it will be particularly useful to collect data outside of the laboratory or clinical setting in the future. This will allow further inferences regarding the pressure changes that occur within a socket not only during various movements, but also during daily living tasks and throughout a longer wearing time. The on-board acceleration measurements could be used to determine compliance, activity level, or to monitor falls, as suggested in literature [42,43]. The temperature sensor may provide insight into the impact of different environments on vacuum performance; inclusion of a second temperature probe could allow differences in internal socket temperature and environmental temperature to be studied. It may also be interesting to integrate our system with additional sensors, such as limb-socket interface pressure sensors or strain gauge sensors to quantify forces and moments applied to the prosthesis and residual limb [43].

Future work may also involve determining correlations between socket air pressure and measurements of the residual limb. For example, residual limb volume loss has been demonstrated in seated [9] and standing [12] tasks. It would be valuable to understand how this loss may be related to average pressures versus cyclical pressure changes, and also to different soft tissue characteristics of residual limbs. It will be valuable to investigate pressure differences between mechanical and electrical pumps, as electrical pumps may be set to greater vacuum pressures than the mechanical pumps evaluated in this study, and will likely generate different pressure profiles. Additionally, future studies should investigate factors that contribute to the effectives of different elevated vacuum systems, such as limb geometry, tissue stiffness, gait and movement strategy, and activity level.

In the future, these techniques should be useful for clinicians, developers, and researchers to address questions related to elevated vacuum system performance. As a clinical tool, this could be used to quickly identify leaks in a socket, understand user compliance, and determine trends in performance throughout the day. There is also a potential for benefit in remote health-care applications [43], or to provide information to developers regarding the impact of various prosthetic components and manufacturing techniques.

## Supporting information

S1 Table Qualitative survey questionnaire. Modified from the OPUS Satisfaction with Device Score (38).
(DOCX)

S2 Table Functional modbility task results. Functional task performance across entire trial, reported as mean ± standard deviation.
(DOCX)

S1 Dataset Supplementary data. (XLSX)
