# Supplementary material for: Real time monitoring of transtibial elevated vacuum prostheses: A case series on socket air pressure
Source: PLoS One. 2018 Oct 22;13(10):e0202716. doi: 10.1371/journal.pone.0202716 (PMC6197629; doi:10.1371/journal.pone.0202716)
Supplement: S1 Table — Modified from the OPUS Satisfaction with Device Score (38). (DOCX) [file pone.0202716.s001.docx]

# S1. Qualitative Survey Questionnaire

**S1 Table. Qualitative survey questionnaire.** Modified from the OPUS Satisfaction with Device Score (38).

|  | 5 (Strongly agree) | 4 (Agree) | 3 (Neither agree nor disagree) | 2 (Disagree) | 1 (Strongly disagree) | N/A |
| --- | --- | --- | --- | --- | --- | --- |
| The prosthesis fits well |  |  |  |  |  |  |
| The prosthesis is comfortable (throughout the testing) |  |  |  |  |  |  |
| My skin/limb feels free of irritation |  |  |  |  |  |  |
| The prosthesis is pain free to wear |  |  |  |  |  |  |
| I feel in control of my prosthesis |  |  |  |  |  |  |
| I feel mobile with my prosthesis on |  |  |  |  |  |  |
| My socket felt secure and did not slip up and down on my limb |  |  |  |  |  |  |
