# Supplementary material for: Real time monitoring of transtibial elevated vacuum prostheses: A case series on socket air pressure
Source: PLoS One. 2018 Oct 22;13(10):e0202716. doi: 10.1371/journal.pone.0202716 (PMC6197629; doi:10.1371/journal.pone.0202716)
Supplement: S2 Table — Functional task performance across entire trial, reported as mean ± standard deviation. (DOCX) [file pone.0202716.s002.docx]

# S2. Functional Mobility Task Results

**S2 Table. Functional modbility task results.** Funcational task performance across entire trial, reported as mean ± standard deviation.

|  |  | **Participant 1** | | **Participant 2** | | **Participant 3** | | **Reported Data** | |
| --- | --- | --- | --- | --- | --- | --- | --- | --- | --- |
|  |  | **Inactive** | **Active** | **Inactive** | **Active** | **Inactive** | **Active** | **Reported** | **Population** |
| 2-Minute Walk | Distance (m) | 178.2 ± 15.5 | 190.1 ± 2.5 | 178.7 ± 6.6 | 186.1 ± 26.7 | 167.5 ± 0.7 | 156.8 | Range: 64.6 to 300.8 Average: 180.9 | Population based sample, N = 1137 (33) |
| 5 Times Sit-to-Stand | Task time (s) | 13.0 ± 1.4 | 13.0 ± 0.0 | 12.0 ± 0.0 | 11.5 ± 0.7 | 17.5 ± 0.7 | 16.0 | Average (60 – 69 y/o): 11.4 | Meta-analysis of normal performance, 13 papers (39) |
| 4-Square Step Test | Task time (s) | 11.4 ± 1.1 | 11.0 ± 0.9 | 10.3 ± 1.0 | 8.3 ± 0.5 | 13.3 ± 0.5 | 13.0 ± 0.8 | Range: 7.4 to 10.0  Average: 8.7 | Normative community dwelling adults, 65 y/o and older, N = 81 (35) |
| L-Test | Task time (s) | 18.3 ± 1.3 | 18.0 ± 0.0 | 18.0 ± 0.8 | 16.8 ± 0.5 | 23.0 ± 1.4 | 23.5 ± 1.0 | Average: 29.5 ± 12.8 | Transtibial amputees, N = 93 (36) |
| Figure-8 Test | # Steps | 12.8 ± 1.0 | 12.5 ± 0.6 | 16.3 ± 1.3 | 15.0 ± 0.8 | 15.0 ± 1.2 | 15.5 ± 0.7 | Range:  16.4 to 18.6  Average: 17.5 | Community dwelling adults with mobility disability, N = 51 (37) |
|  | Task time (s) | 7.8 ± 1.0 | 7.3 ± 0.5 | 7.8 ± 1.0 | 6.8 ± 1.0 | 9.8 ± 1.0 | 10.0 ± 0.0 | Range: 9.8 to 11.2  Average: 10.5 |  |
